# Calreticulin increases growth and progression of natural killer/T-cell lymphoma

**DOI:** 10.18632/aging.104030

**Published:** 2020-11-18

**Authors:** Yan Zheng, Chuntuan Li, Pengliang Xin, Qunyi Peng, Weiyu Zhang, Shengquan Liu, Xiongpeng Zhu

**Affiliations:** 1 Department of Hematology, The First Hospital of Quanzhou Affiliated to Fujian Medical University, Quanzhou 362000, Fujian Province, China; 2 Department of Pathology, The First Hospital of Quanzhou Affiliated to Fujian Medical University, Quanzhou 362000, Fujian Province, China

**Keywords:** natural killer/T-cell lymphoma, calreticulin, growth, migration, epithelial-mesenchymal transition

## Abstract

In this study, we investigated the role of calreticulin (CALR) in the pathogenesis of natural killer/T-cell lymphoma (NKTCL). CALR expression was significantly higher in the NKTCL tissues than normal control tissues in the GSE80632 dataset. High CALR expression correlated with poorer overall survival of NKTCL patients (*P* = 0.0248). CALR mRNA and protein levels were significantly higher in NKTCL cell lines (NK92, SNK6, and SNT8) than normal NK cells. CALR-silenced SNK6 cells generated significantly smaller xenograft tumors in immunodeficient NCG mice than control SNK6 cells. CALR-knockdown NKTCL cells showed significantly less *in vitro* proliferation and Transwell migration than the controls. CALR knockdown inhibited G1-to-S phase cell cycle progression by increasing the levels of p27 cell cycle inhibitor and reducing the levels of cyclin E2 and cyclin-dependent kinase 2 (CDK2). CALR knockdown inhibited epithelial-to-mesenchymal transition (EMT) by decreasing the levels of β-catenin and TCF/ZEB1 and upregulating E-cadherin. These data demonstrate that CALR regulates the growth and progression of NKTCL cells by modulating G1-to-S cell cycle progression and EMT.

## INTRODUCTION

Natural killer T-cell lymphoma (NKTCL) is an aggressive type of non-Hodgkin lymphoma (NHL) that is associated with poor survival outcomes [1]. NKTCL is rare in Europe and North America, but highly prevalent in Asia and Latin American countries [2]. Although the exact pathogenesis of NKTCL is unknown, the aggressive forms of this tumor are associated with Epstein-Barr virus (EBV) infections [3]. There is no consensus on the standardized therapy for NKTCL patients, and the treatment strategy is mainly based on the disease stage of the patients at diagnosis [4]. Currently, the therapeutic management of NKTCL mostly involves radiotherapy and chemotherapy, although targeted therapy and stem cell transplantation have shown great promise in a few cases [4–7]. However, the prognosis for patients with advanced-stages of NKTCL remains extremely poor because of the highly aggressive nature of the tumor and a high frequency of chemotherapy resistance [1].

L-asparaginase-containing treatment regimens are currently the first-line of therapy for patients with advanced-stage or disseminated NKTCL [8–10]. L-asparaginase-based regimens achieve satisfactory therapeutic outcomes in patients with advanced-stage, refractory, or relapsed NKTCL [11–13]. However, a significant proportion of patients with NKTCL are associated with disease progression or relapse after initial treatment [14]. In a multicenter retrospective study involving 179 patients with refractory and relapsed NKTCL that were treated with L-asparaginase-based regimens, the median second progression-free survival (PFS) was 4.1 months and the median overall survival (OS) was 6.4 months [15]. Hence, there is an urgent need for novel treatments with high efficacy and low toxicity for patients with refractory or relapsed NKTCL.

Calreticulin (CALR) is a 46-kDa Ca^2+^-binding chaperone that resides predominantly in the lumen of the endoplasmic reticulum [16]. Previous studies indicate that CALR plays a role in autoimmunity, wound healing, cell adhesion, gene expression, Ca^2+^ storage and signaling, and lectin-like chaperone functions [17]. Several studies have also implicated CALR in oncogenesis, including esophageal squamous cell carcinoma [18, 19], gastric cancer [20], bladder cancer [21], breast cancer [22], pancreatic cancer [23], hepatocellular carcinoma [24], prostate cancer [25], ovarian cancer [26] and glioma [27]. However, the role of CALR in NKTCL is not known. Therefore, we investigated the mechanistic role of CALR in the growth and progression of NKTCL using both *in vitro* and *in vivo* models.

## RESULTS

### High CALR expression correlates with poorer overall survival in NKTCL patients

**Figure 1:**
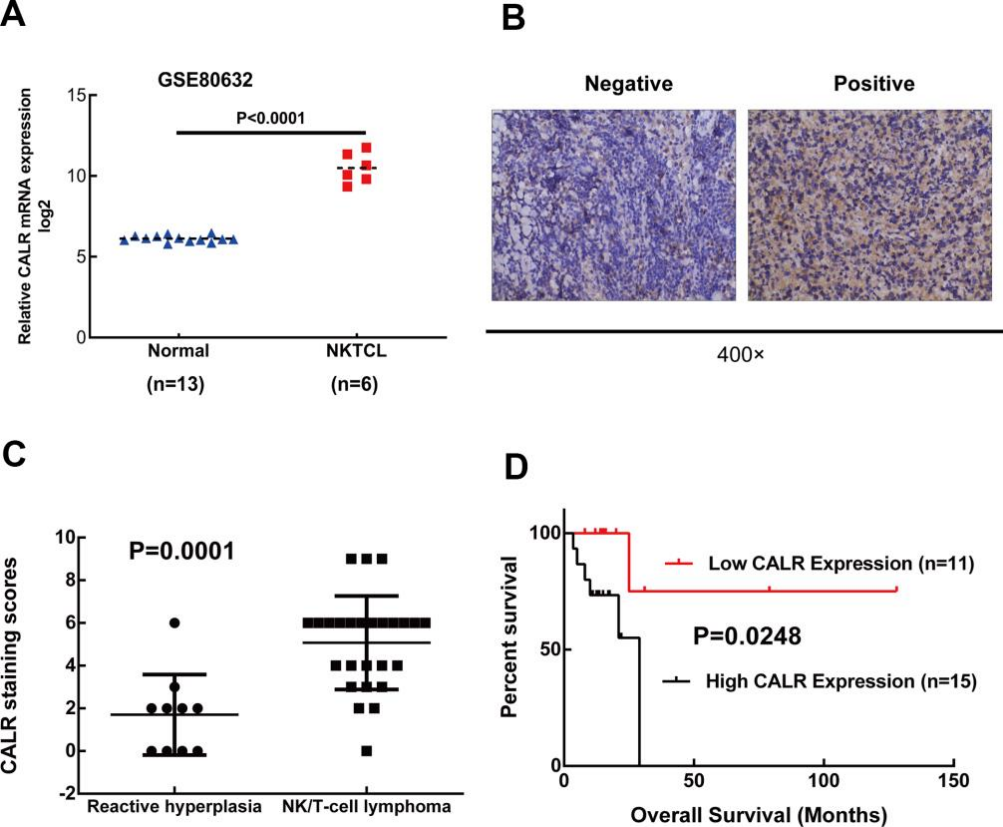
**High CALR expression correlates with shorter overall survival in patients with NKTCL.** (**A**) CALR mRNA expression is upregulated in the NKTCL specimens compared to normal control tissues based on the GSE80632 dataset analysis (*P* < 0.05 according to Mann-Whitney *U* test). Note: The error bars represent standard error. (**B**) Representative images (400X magnification) show positive and negative immunohistochemical staining of CALR in the 26 NKTCL patient tissues and 10 reactive lymphoid hyperplasia patient tissues (controls). (**C**) Quantitative analysis based on immunohistochemical staining shows CALR protein levels in the 26 NKTCL and 10 reactive lymphoid hyperplasia patient samples. (**D**) Kaplan-Meier survival curve analysis shows the overall survival of NKTCL patients with high and low CALR expression. As shown, high CALR expression correlates with shorter overall survival in NKTCL patients.

RNA-seq data analysis of the GSE80632 dataset showed that CALR expression was significantly upregulated in 6 NKTCL tissues compared to 13 normal control tissues (*P* < 0.001, Figure 1A). IHC analysis of 26 NKTCL patients and 10 patients with reactive lymphoid hyperplasia (controls) showed that CALR was predominantly localized in the cytoplasm of NKTCL cells (Figure 1B). Moreover, higher CALR protein expression was detected in a larger proportion of NKTCL patient specimens compared to the reactive lymphoid hyperplasia patient specimens (11/26 vs. 1/10, *P* = 0.0001; Figure 1C). These data suggest that CALR expression is significantly upregulated in NKTCL.

Next, we analyzed the association between CALR expression and the clinicopathological characteristics of patients with NKTCL. We divided the 26 NKTCL patients into low (immunoreactivity score: 0–3) or high (immunoreactivity score: ≥4) CALR expression groups. IHC data showed that 11/26 (42.3%) NKTCL patients showed low CALR expression and 15/26 (57.7%) NKTCL patients showed high CALR expression. Moreover, high CALR expression was negatively associated with age (*P* < 0.001), but did not show any correlation with gender, serum LDH, B symptoms, ECOG performance status, EBV DNA load, bone marrow invasion or PINK. Furthermore, CALR expression was higher in patients with stage III/IV NKTCL compared to those with stage I/II NKTCL (*P* = 0.105). The 26 NKTCL patients had all received L-asparaginase-based chemotherapy alone or in combination with radiotherapy; two patients also received autologous hematopoietic stem cell transplantation. The initial therapy achieved complete response in 18 cases and incomplete response in 8 cases. Based on immunohistochemical staining, among the 18 cases with complete response, 13 showed low CALR expression (72.22%) and 5 showed high CALR expression (27.78%). On the other hand, among the 8 cases with incomplete response, 4 showed low CALR expression (50%) and 4 showed high CALR expression (50%). However, CALR expression levels between patients with complete and incomplete responses were statistically insignificant (*P* = 0.382, Table 1). Kaplan-Meier survival curve analysis showed that patients with high CALR expression correlated with poorer OS (*P* = 0.0248, Figure 1D). Overall, these data suggest that higher CALR expression correlates with poorer prognosis of NKTCL patients.

**Table 1:** Associations of CALR expression with the clinicopathological characteristics in NKTCL patients.

**Characteristics**	**No. patients (*n* = 26)**	**CALR**		***P* value**
		**Low expression (*n* = 11)**	**High expression (*n* = 15)**	
Age (years)				
≤ 60	16	4	12	0.024
> 60	10	7	3	
Gender				
Male	15	6	9	0.781
Female	11	5	6	
ECOG performance status				
0/1	23	10	13	0.738
≥ 2	3	1	2	
Serum LDH				
Normal	22	10	12	0.446
Increased	4	1	3	
B symptoms				
Absence	17	7	10	0.873
Presence	9	4	5	
Bone marrow involvement				
No	25	11	14	0.382
Yes	1	0	1	
Hemophagocytosis				
No	25	11	14	0.382
Yes	1	0	1	
EBV DNA load				
Less than detected	13	6	7	0.691
Detected	13	5	8	
Ann Arbor stage				
I/II	18	9	9	0.105
III/IV	8	2	6	
PINK				
Low/Intermediate	23	9	14	0.145
High	3	2	1	
Treatment response				
CR	18	13	5	0.382
non-CR	8	4	4	

### CALR expression is upregulated in human NKTCL cell lines

**Figure 2:**
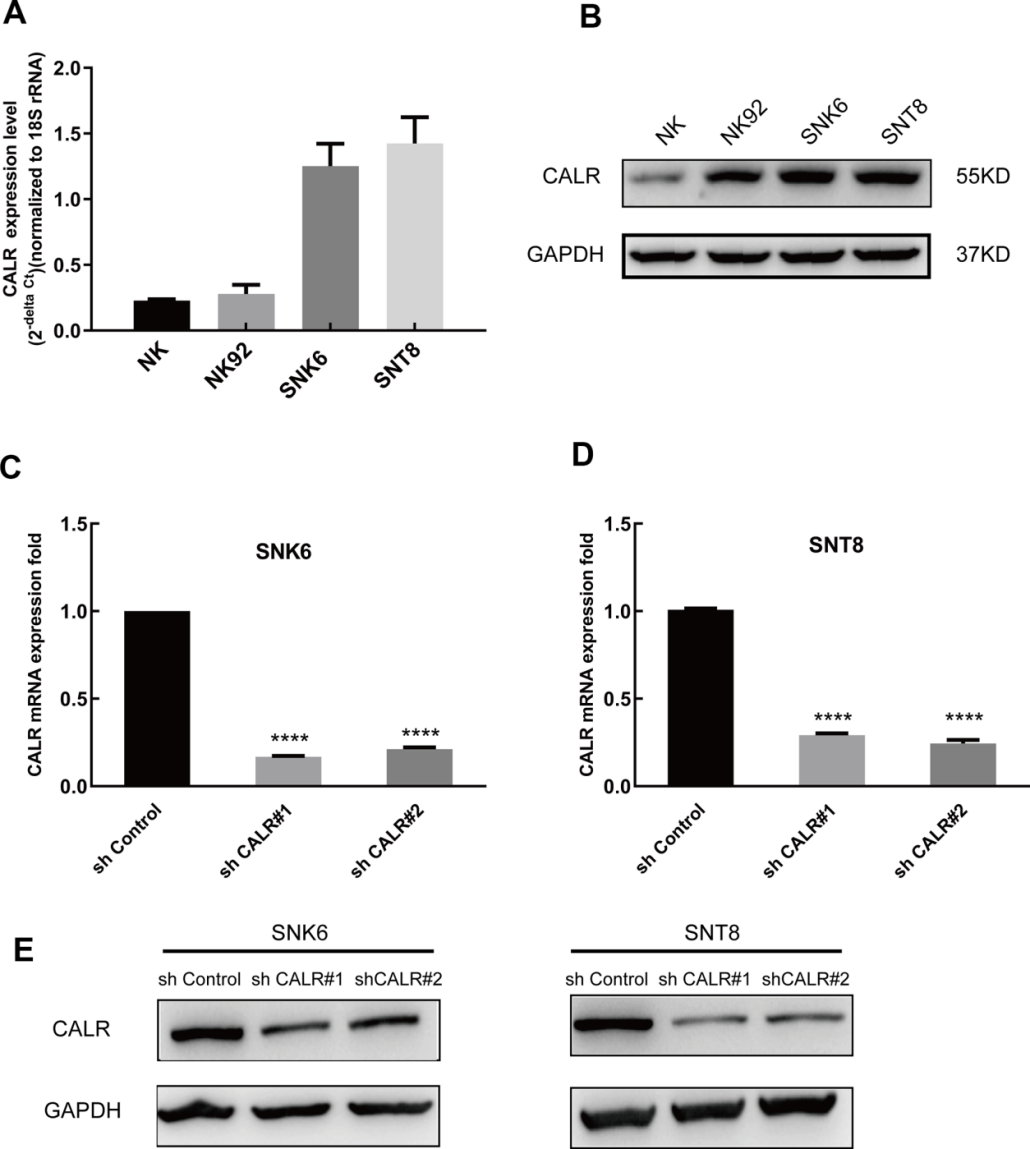
**CALR expression is upregulated in the NKTCL cell lines.** (**A**) Quantitative real time PCR analysis shows the CALR mRNA levels relative to 18S rRNA levels in the NKTCL cell lines (NK92, SNK6 and SNT8) and the normal NK cells. (**B**) Western blot analysis shows the CALR protein levels in the NKTCL cell lines (NK92, SNK6 and SNT8) and the normal NK cells. (**C**) Q-PCR analysis shows the CALR mRNA levels relative to 18S rRNA levels in the SNK6 cells transfected with shCALR#1, shCALR#2, shCALR#3, and shControl. (**D**) Q-PCR analysis shows the CALR mRNA levels relative to 18S rRNA levels in the SNT8 cells transfected with shCALR#1, shCALR#2, shCALR#3, and shControl. (**E**) Western blot analysis shows the CALR protein expression in SNK6 or SNT8 cells transfected with shCALR#1, shCALR#2, shCALR#3, and shControl. Note: **** *P* < 0.0001.

Quantitative real time PCR and western blotting analysis showed that CALR mRNA and protein levels were significantly higher in the NKTCL cell lines, NK92, SNK6, and SNT8 compared to the normal NK cells (Figure 2A, 2B). Since CALR expression was higher in the SNK6 and SNT8 cells compared to the NK92 cells, we selected SNK6 and SNT8 cells for the subsequent experiments.

We then designed 3 CALR-targeting shRNAs and established stable CALR-knockdown SNK6 and SNT8 cell lines. QPCR and western blotting assays showed that CALR mRNA and protein levels were significantly reduced in the SNK6 and SNT8 cell lines transfected with shCALR #1 and shCALR #2 compared to those transfected with shCALR #3 (Figure 2C–2E). Hence, we chose SNK6 and SNT8 cell lines transfected with shCALR #1 and shCALR #2 for further experiments.

### CALR knockdown suppresses *in vitro* and *in vivo* growth of NKTCL cells

**Figure 3:**
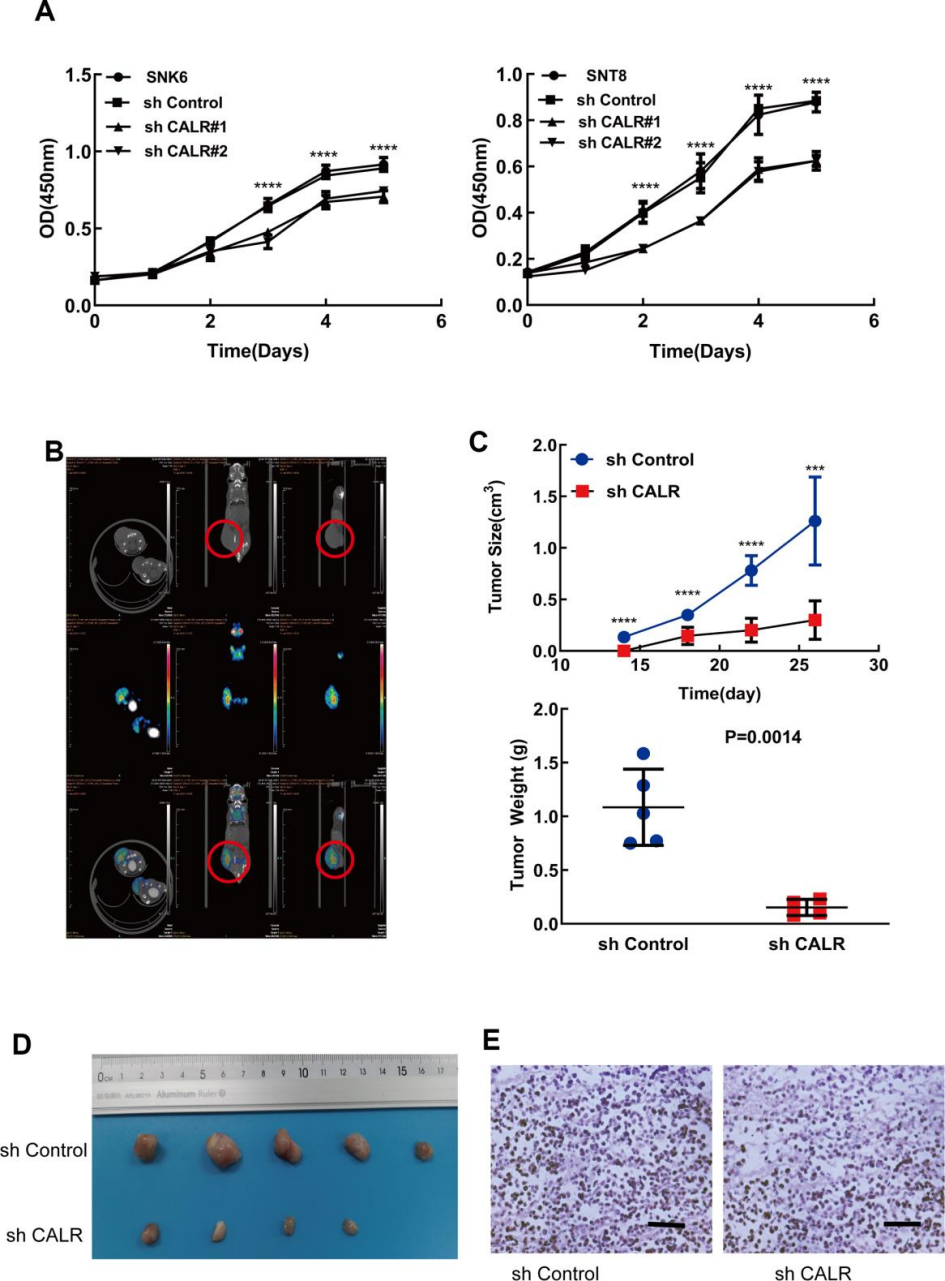
**CALR knockdown suppresses *in vitro* and *in vivo* NKTCL cell growth.** (**A**) CCK-8 assay results show the viability of SNK6 and SNT8 cells transfected with shCALR #1, shCALR #2 or shControl. (**B**) Representative images show the xenograft tumors at 14 days in mice subcutaneously injected with the shControl-transfected SNK6 cells. The red circles indicate visible tumors. (**C**, **D**) Xenograft tumor growth analysis shows the tumor volume and weight in mice subcutaneously injected with CALR knockdown SNK6 cells in comparison with those injected with shControl-transfected SNK6 cells. Knockdown of CALR inhibited tumor formation of the SNK6 cells *in vivo* (**C**, upper panel and **D**). The tumor weight was significantly reduced in the CALR-silenced group compared to the control group (**C**, lower panel). (**E**) Representative H&E stained sections show the morphology of xenograft tumors derived from mice injected subcutaneously with shControl or CALR-knockdown SNK6 cells (bar=200μm). Note: The data are shown as means ± standard deviation; **** *P* < 0.0001, *** *P* < 0.001; OD = optical density.

Next, we analyzed the effects of CALR knockdown on the proliferation of NKTCL cells. CCK-8 assay analysis showed that the proliferation of CALR-knockdown SNK6 and SNT8 cells was significantly reduced compared to the control SNK6 and SNT8 cells (Figure 3A). This suggests that CALR promotes NKTCL cell proliferation.

We then investigated the *in vivo* effects of CALR silencing in NKTCL cells by subcutaneously injecting shControl-transfected or sh-CALR-transfected SNK6 cells into the NCG mice. Xenograft tumors were visible in the mice injected with the shControl-transfected SNK6 cells (control group) within 14 days post-injection compared to 21 days for those injected with the sh-CALR-transfected SNK6 cells or CALR-silenced group (Figure 3B). Moreover, the tumor volume was significantly reduced in the CALR-silenced group compared to the control group (Figure 3C, 3D). H&E staining data showed comparable histological features between the tumors in the CALR-silenced and the control groups (Figure 3E). Taken together, these results demonstrate that CALR knockdown inhibits *in vivo* proliferation of NKTCL cells.

### CALR knockdown induces G1-S cell cycle arrest in NKTCL cells

**Figure 4:**
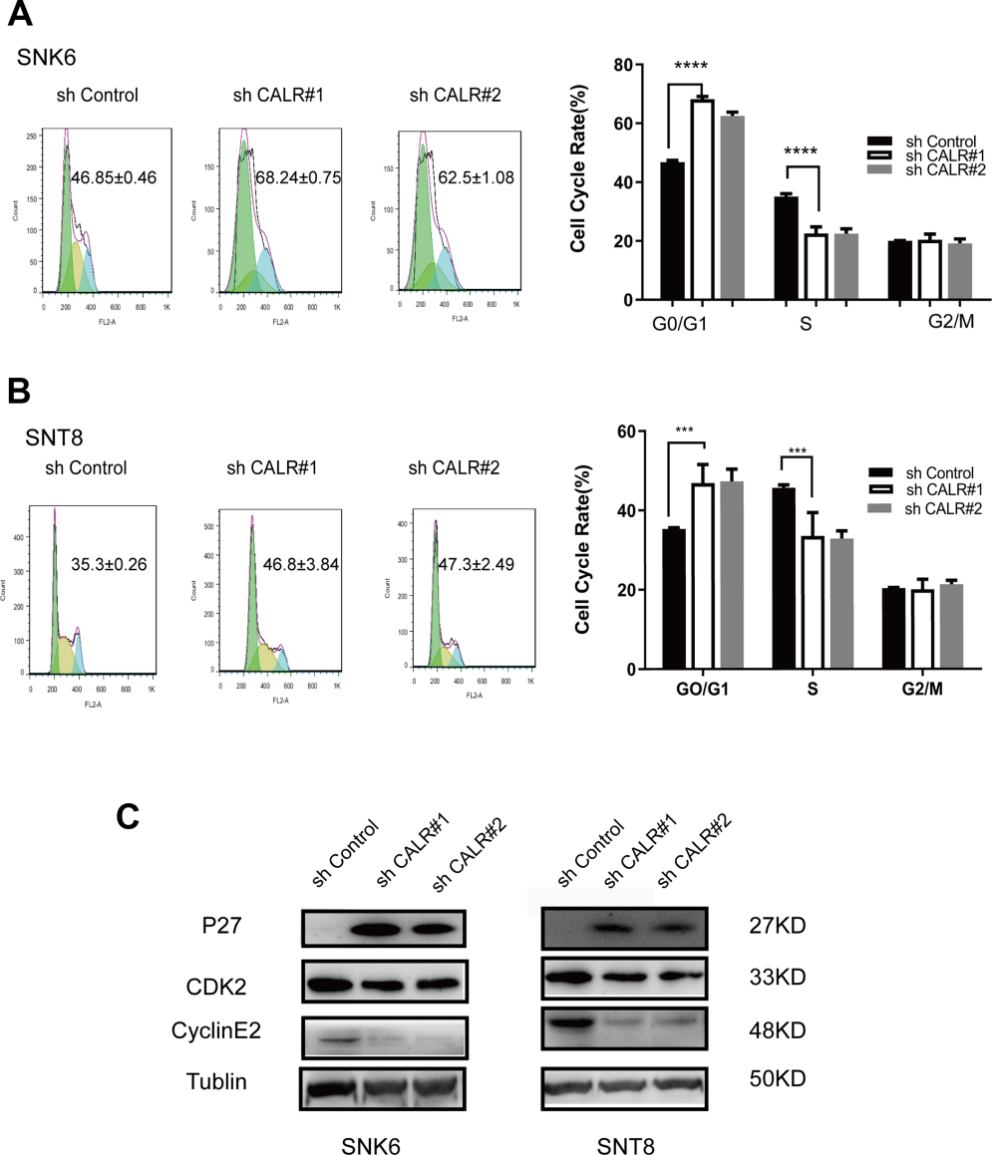
**CALR knockdown inhibits G1 to S-phase cell cycle transition in NKTCL cell lines.** (**A**, **B**) Flow cytometry analysis shows the proportions of G0/G1 phase and S-phase cells in the control and CALR-knockdown SNK6 and SNT8 cell lines. The cells were stained with PI. (**C**) Western blot analysis shows the expression of cell cycle regulatory proteins, P27, CDK2 and Cyclin E2 in the control and CALR-knockdown SNK6 and SNT8 cell lines. Note: All experiments were performed in triplicates; *P* values were analyzed using ANOVA; **** *P* < 0.0001, *** *P* < 0.001.

Flow cytometry analysis showed higher proportion of G0/G1 phase cells and lower proportion of S-phase cells in the CALR-knockdown SNK6 and SNT8 cells compared to the corresponding controls (Figure 4A, 4B). This suggests that CALR knockdown inhibits G1-S phase cell cycle transition. We then analyzed the expression of key regulators of the G1-S phase transition by western blotting. The levels of p27 cell cycle inhibitor were significantly upregulated and the levels of cyclin E2 and cyclin-dependent kinase 2 (CDK2) were significantly reduced in the CALR-knockdown SNK6 and SNT8 cells compared to the corresponding controls (Figure 4C). These findings suggest that CALR knockdown suppresses NKTCL cell proliferation by inhibiting G1-S cell-cycle progression.

### CALR knockdown inhibits NKTCL cell migration through modulating EMT

**Figure 5:**
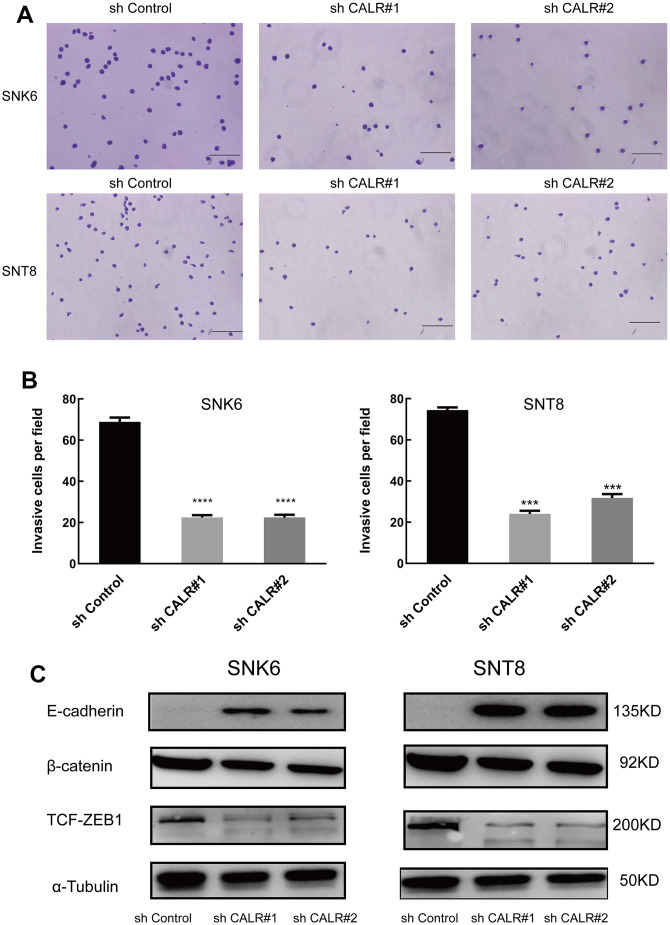
**CALR knockdown suppresses *in vitro* transwell migration and EMT in NKTCL cells.** (**A**, **B**) Transwell migration assay results show the numbers of migrating cells in the control and CALR-knockdown SNK6 and SNT8 cells (bar=200μm). Note: The data are shown as means ± standard deviation; **** *P* < 0.0001, *** *P* < 0.001. (**C**) Western blot analysis shows the expression of EMT-related proteins, E-cadherin, β-catenin, and TCF-ZEB1, in the control and CALR-knockdown SNK6 and SNT8 cells, and α-tubulin was used as the loading control.

We then performed the Transwell migration assays to determine the effects of CALR knockdown on the migration of NKTCL cells. The numbers of migrating CALR-knockdown SNK6 or SNT8 cells were significantly reduced compared to the corresponding controls (Figure 5A, 5B).

CALR promotes TGFβ-induced epithelial-mesenchymal transition (EMT) and subsequent cardiomyogenesis during the differentiation of embryoid bodies derived from the mouse embryonic stem cells [28]. Therefore, we analyzed the expression of key biomarkers related to EMT. Western blotting analysis showed that β-catenin and TCF/ZEB1 protein levels were significantly lower and E-cadherin levels were significantly higher in the CALR-knockdown SNK6 or SNT8 cells compared to the corresponding controls (Figure 5C). This suggests that CALR knockdown suppresses NKTCL cell migration by inhibiting EMT.

## DISCUSSION

NKTCL is a rare but highly aggressive malignancy associated with poor prognosis [1]. Although great advances have been made in the treatment of NKTCL, especially with the availability of highly effective L-asparaginase-containing regimens, the clinical outcomes of NKTCL remain unsatisfactory [5]. Therefore, novel treatments are necessary to improve the survival outcomes of NKTCL patients.

The pathogenesis of NKTCL is well understood based on the knowledge of the gene expression profiles and the status of signaling pathways in NKTCL patient tissue samples [29, 30]. CALR protein modulates diverse cellular processes, such as keratinocyte differentiation, embryonic stem cell differentiation, and tumorigenesis [16, 17, 31–33]. In this study, we demonstrate that CALR expression is significantly upregulated in the NKTCL tissues compared to the reactive lymphoid hyperplasia tissues. Moreover, CALR expression is upregulated in the NKTCL cell lines compared to the normal NK cells. Furthermore, CALR knockdown inhibits NKTCL cell growth, migration, and cell-cycle progression. Collectively, these data demonstrate show that CALR promotes growth and progression of NKTCL. Overall, our findings suggest that CALR is a potential therapeutic target for NKTCL.

Previous studies have shown that CALR is a prognostic factor for several human cancers [34, 35]. Xu and colleagues reported that low CALR expression was significantly associated with endometrial cancer progression and poor prognosis [34]. In gastric cancer patients, positive immunohistochemical staining of CALR is associated with poor overall survival; moreover CALR is an independent prognostic indicator for the survival of gastric cancer patients [35]. Colon cancer patients with high CALR expression are associated with a higher 5-year survival rate (43% vs. 16%) compared to those with low CALR expression [36]. Renal cancer patients with higher CALR expression show a lower 5-year survival rate (44% vs. 76%) compared to those with lower CALR expression [37]. In this study, Kaplan-Meier analysis demonstrates that high CALR expression is associated with poorer OS in patients with NKTCL. This suggests that CALR is a potential prognostic biomarker for patients with NKTCL.

Previous studies have demonstrated that CALR is an oncogenic protein that plays a critical role in cell migration, invasion and metastasis [38, 39]. EMT plays a critical role in cancer progression and metastasis [40]. CALR silencing inhibits TGF-β1-induced suppression of E-cadherin, thereby decreasing the invasiveness and migration of gastric cancer cells [41]. Moreover, CALR promotes epidermal growth factor (EGF)-triggered EMT via the Integrin/EGFR/ERK/MAPK pathway and alters intracellular Ca^2+^ levels in the pancreatic cancer cells [42]. In lung cancer, CALR mediates TGF-β1-induced EMT by modulating the Smad and calcium signaling pathways [43]. In this study, we demonstrate that CALR knockdown reduces the numbers of migrating NKTCL cells in the Transwell migration assay by downregulating β-catenin and TCF/ZEB1 expression and upregulating E-cadherin expression. These data suggest that CALR knockdown suppresses NKTCL cell migration by inhibiting EMT.

In summary, our study demonstrates that CALR promotes *in vivo* and *in vitro* growth and progression of NKTCL cells. We demonstrate that CALR knockdown suppresses NKTCL cell growth and migration by inducing G1-S cell cycle arrest and inhibiting EMT. Therefore, CALR is a potential prognostic biomarker and therapeutic target for NKTCL, but further studies are necessary to establish the clinical significance of CALR.

## MATERIALS AND METHODS

### NKTCL cell lines and cell culture

The human natural killer NK-92 cell line was purchased from the China Center for Type Culture Collection (Cat. no. GDC052; Wuhan, China), and grown in α-MEM medium (Cat. No.12571089; Life Technologies, Carlsbad, CA, USA) supplemented with 12.5% fetal bovine serum (FBS; Cat. No.1614007; Gibco, Grand Island, NY, USA), 12.5% horse serum (Cat. No. 26050070; Gibco), 1.5 g/L sodium bicarbonate, 2 mM L-glutamine (Cat. No. 25030149; Gibco), 100 to 200 U/ml recombinant IL-2 (Cat. No. 200-02; PeproTech, Rocky Hill, NJ, USA,), 0.1 mM 2-mercaptoethanol, 0.2 mM inositol (Cat. No. I5125; Sigma-Aldrich, St. Louis, MO, USA), 0.02 mM folic acid, and 1% penicillin-streptomycin solution (Cat. No. SV30010; Solarbio, Beijing, China).

The human NKTCL SNK6 and SNT8 cell lines were obtained from the Shanghai Yubo Biological Technology Co., Ltd. (Cat. No. YB-H3256; Shanghai, China), and grown in RPMI-1640 medium (Cat. No. A4192301; Gibco), supplemented with 10% FBS (Cat. No. SH30396.03; Hyclone, Logan, UT, USA), and 1% penicillin-streptomycin solution. The cells were grown in a humidified chamber at 37° C and 5% CO_2_.

We harvested human NK cells from healthy donors with the Ficoll-Paque gradient and purified them by negative magnetic selection using the human NK cell isolation kit (Cat. No.130-092-657; Miltenyi Biotec, Inc., Cambridge, MA, USA) according to the manufacturer’s instructions. The purity of the NK cell preparations was determined by flow cytometry, and >95% pure NK cells were used for subsequent experiments.

### Human NKTCL and control tissue specimens

We obtained twenty-six formalin-fixed paraffin-embedded human NKTCL specimens and 10 reactive lymphoid hyperplasia specimens as controls from the Quanzhou First Hospital (Quanzhou, China). These samples were collected from patients between April 2008 and April 2017. The demographics of the study subjects and their clinical characteristics including age, gender, symptoms, Eastern Cooperative Oncology Group (ECOG) performance status, Ann Arbor stage, bone marrow involvement, hemophagocytosis, serum lactate dehydrogenase (LDH) level, EBV DNA load, and prognostic index of natural killer cell lymphoma (PINK) and response to primary therapy were obtained from the medical records.

### Immunohistochemistry (IHC)

We performed IHC analysis to determine CALR expression in the formalin-fixed paraffin-embedded human NKTCL and reactive lymphoid hyperplasia specimens. Briefly, the 4-μm thick specimens were deparaffinized with xylene, rehydrated, and incubated with 3% hydrogen peroxide to block endogenous peroxidase. Then, the sections were incubated overnight at 4° C with the monoclonal rabbit anti-CALR antibody (1:200 dilution; Cat. No. 12238; Cell Signaling Technology, Beverly, MA, USA). Then, the samples were incubated with the biotinylated anti-rabbit secondary antibody (1:150 dilution; Cat. No.14708; Cell Signaling Technology) for 1 h at room temperature followed by DAB staining (Cat. No. P0203; Beyotime, Shanghai, China). The samples were counterstained with hematoxylin and eosin (HE). The sections were examined under the inverted microscope (TE2000 –U, Nikon, Japan) and scored as previously described [44].

### CALR gene expression data analysis

The gene expression data from the GSE80632 dataset was downloaded from the GEO database (https://www.ncbi.nlm.nih.gov/gds/?term=) and CALR expression was analyzed by searching for LIMN-1736256, the ID for CALR. The expression of CALR RNA was log-transformed (log2) and the data was analyzed using the unpaired student's *t* test.

### Cell transfection

We purchased short-hairpin RNA targeting CALR (shCALR#1: CCAGTATCTATGCCTATGATA; shCALR#2: GCACGGAGACTCAGAATACAA, and shCALR#3: CCACCCAAGAAGATAAAGGAT) and the negative control shRNA (shControl) from the Sangon Biotech (Shanghai) Co., Ltd. (Shanghai, China). The shRNAs were cloned into the pLKO.1-puro vector (gift from Dr. Ruian Xu from the School of Biomedical Sciences, Huaqiao University). The human embryonic kidney 293FT cells were transiently transfected with the recombinant lentiviral shRNA constructs along with the pCMVΔ8.9 and pcDNA-VSVG plasmids (gifts from Dr. Ruian Xu from the School of Biomedical Sciences, Huaqiao University) in a ratio of 2:2:1 using Lipofectamine 3000 (Cat. No. L30000015; Invitrogen, Carlsbad, CA, USA) according to the manufacturer’s instructions. The culture supernatant was collected after 3 days, and the viral particles were concentrated by centrifugation at 75,600 ×*g* at 4° C. Then, the SNK6 and SNT8 cell lines were infected with the lentiviruses carrying shCALR#1, shCALR#2, shCALR#3 or sh-Control in the presence of 9 μg/mL polybrene (Cat. No. 41639; Sigma-Aldrich). The positive clones were selected with medium containing 1 μg/ml puromycin (Cat. No. P8833; Sigma-Aldrich). The efficiency of the CALR knockdown by shRNAs was analyzed by western blotting and quantitative real-time PCR (qRT-PCR) assays.

### Quantitative real time PCR

Total RNA was extracted using Trizol (Cat. No.15596-026; Invitrogen, Carlsbad, CA, USA) according to the manufacturer’s instructions, and reverse transcribed into cDNA using the HiScript® II 1^st^ Strand cDNA Synthesis Kit (Cat. No. R212-02; Vazyme Biotech, Piscataway, NJ, USA). Then, the qPCR assay was performed using CALR-specific primers (sense, 5′- AACCCGTTGAACCCCATT-3′; antisense, 5′-CCATCCAATCGGTAGTAGCG-3′) with the SYBR Green PCR Master Mix (Cat. No. Q111-02; Vazyme Biotech) in an ABI-7500 Fast Real-Time PCR system (Applied Biosystem; Foster City, CA, USA). The 18S rRNA was used as the internal control. Relative gene expression was calculated using the 2^-ΔΔCt^ method.

### Western blotting

Total cellular proteins were extracted using the RIPA buffer and the cell lysates were centrifuged at 14,000 r/min and 4° C for 10 mins. The protein concentration was quantified using the BCA Protein Assay (Cat. No. P0010; Beyotime, Shanghai, China). Equal amounts of total protein lysates were separated on a 10% SDS-PAGE, and the separated proteins were electrotransferred onto 0.2 μm PVDF membranes. Then, the membranes were incubated overnight at 4° C with the primary monoclonal rabbit anti-CALR antibody (1:1,000; Cat. No. 12238; Cell Signaling Technology), primary antibodies in the epithelial-mesenchymal transition (EMT) antibody sampler kit (Cat No. 9782; Cell signaling Technology) and the cell cycle regulation antibody sampler kit (Cat No. 9932; Cell signaling Technology), primary mouse anti-GAPDH antibody (1:1,000, Cat. No. 97166; Cell signaling Technology), and the anti-α-tubulin antibody (Beyotime, Shanghai, China). Then, the blots were probed with the HRP-conjugated secondary antibodies. The blots were developed using ECL, photographed and quantified using the Image J software.

### CCK-8 cell viability assay

Cell viability was measured using the Cell Counting Kit-8 (CCK-8, Cat. No. C0038; Beyotime) according to the manufacturer’s instructions.

### Flow cytometry analysis of cell cycle

Cell cycle analysis was performed by flow cytometry using propidium iodide (PI). Briefly, the cells were washed with 1X PBS, fixed in pre-cooled 75% ethanol at 4° C overnight, washed with ice-cold PBS, and re-suspended in 0.2 to 0.5 mg/mL RNase A (Cat. No. R4875; Sigma-Aldrich) at 37° C for 30 mins. Then, the cells were then incubated with 30 μg/mL PI (Cat. No. P4170; Sigma-Aldrich) in the dark at 4° C for 30 min. The cell viability was evaluated by flow cytometry using a FACSCanto II Flow Cytometer (BD Biosciences; Bedford, MA, USA) and the percentage of PI-positive cells were analyzed from the FACS plots.

### Transwell cell migration assay

Cell migration was analyzed using the 24-well Transwell plates (Cat. no. 3422; Corning Inc., Corning, NY, USA,) with 8 μm pore filters. We seeded 2 × 10^5^ serum-starved cells in 200 μL of serum-free RPMI-1640 medium into the upper chamber and 600 μL of RPMI-1640 medium supplemented with 10% FBS into the lower chamber and incubated the plate in a humidified chamber at 37° C and 5% CO_2_. After 24 h, the cells that had migrated through the basement membrane layer were fixed with 4% PFA, stained with 0.1% crystal violet, and counted under a light microscope at a high magnification in at least 3 fields.

### *In vivo* tumor xenograft growth assay

Six- to eight-week old female immunodeficient NCG mice were purchased from the Nanjing Biomedical Research Institute of Nanjing University (Nanjing, China), housed in pathogen-free conditions, and given free access to clean food and water. We subcutaneously injected 2 × 10^6^ control or CALR-knockdown SNT6 cells into the flanks of NCG mice (n=5 per group). The tumor size was measured twice a week using a vernier calipers and the tumor volume (*V*) was calculated as *V* = (*L* × W^2^)/2, where *L* indicates the tumor length, and *W* indicates the tumor width. The mice were sacrificed at 26 days after injecting the cells subcutaneously. The subcutaneous xenograft tumors were harvested, weighed, and examined by HE staining.

### Statistical analysis

The data are expressed as means ± standard deviation (SD). All statistical analyses were performed using the SPSS version 22.0 statistical software (SPSS, Inc.; Chicago, IL, USA) and GraphPad Prism version 6 (GraphPad Software; La Jolla, CA, USA). We performed Kaplan-Meier survival analysis and estimated overall survival (OS) as the duration from the initial diagnosis to the date of last follow-up or death. The associations between CALR expression and the clinicopathological characteristics were evaluated using the Fisher’s exact test. *P* < 0.05 was considered statistically significant.

### Ethical statement

This study was approved by the Ethics Review Committee of Quanzhou First Hospital Affiliated to Fujian Medical University. The protocols for the animal experiments were performed according to the guidelines for the care and use of laboratory animals as approved by the Ethics Review Committee of Huaqiao University. All experimental procedures were performed in accordance with the Declaration of Helsinki. We obtained written informed consent from all the study participants.
